# Antimicrobial resistance patterns in *Streptococcus dysgalactiae* in a One Health perspective

**DOI:** 10.3389/fmicb.2024.1423762

**Published:** 2024-06-06

**Authors:** Marte Glambek, Steinar Skrede, Audun Sivertsen, Bård Reiakvam Kittang, Alba Kaci, Christine Monceyron Jonassen, Hannah Joan Jørgensen, Norwegian Study Group on Streptococcus dysgalactiae, Oddvar Oppegaard

**Affiliations:** ^1^Department of Medicine, Haukeland University Hospital, Bergen, Norway; ^2^Department of Clinical Medicine 2, Department of Clinical Science, University of Bergen, Bergen, Norway; ^3^Department of Microbiology, Haukeland University Hospital, Bergen, Norway; ^4^Department of Internal Medicine, Haraldsplass Deaconess Hospital (HDS), Bergen, Norway; ^5^Center for Laboratory Medicine, Østfold Hospital, Grålum, Norway; ^6^Department of Virology, Division of Infection Control and Environmental Health, Norwegian Institute of Public Health, Oslo, Norway; ^7^Norwegian Veterinary Institute, Ås, Norway

**Keywords:** *Streptococcus dysgalactiae*, antibiotic resistance, One Health, whole genomic sequencing, horizontal genetic transfer, animal, human, Norway

## Abstract

**Background:**

*Streptococcus dysgalactiae* (SD) is an important pathogen in humans as well as in a broad range of animal species. Escalating rates of antibiotic resistance in SD has been reported in both human and veterinary clinical practice, but the dissemination of resistance determinants has so far never been examined in a One Health Perspective. We wanted to explore the occurrence of zoonotic transmission of SD and the potential for exchange of resistance traits between SD from different host populations.

**Methods:**

We compared whole genome sequences and phenotypical antimicrobial susceptibility of 407 SD isolates, comprising all isolates obtained from human bloodstream infections in 2018 (*n* = 274) and available isolates associated with animal infections from the years 2018 and 2019 (*n* = 133) in Norway.

**Results:**

Antimicrobial resistance genes were detected in 70 (26%), 9 (25%) and 2 (2%) of the isolates derived from humans, companion animals and livestock, respectively. Notably, distinct host associated genotypic resistomes were observed. The *erm*(A) gene was the dominant cause of erythromycin resistance in human associated isolates, whereas only *erm*(B) and *lsa*(C) were identified in SD isolates from animals. Moreover, the tetracycline resistance gene *tet*(O) was located on different mobile genetic elements in SD from humans and animals. Evidence of niche specialization was also evident in the phylogenetic analysis, as the isolates could be almost perfectly delineated in accordance with host species. Nevertheless, near identical mobile genetic elements were observed in four isolates from different host species including one human, implying potential transmission of antibiotic resistance between different environments.

**Conclusion:**

We found a phylogenetic delineation of SD strains in line with host adapted populations and niche specialization. Direct transmission of strains or genetic elements carrying resistance genes between SD from different ecological niches appears to be rare in our geographical region.

## Introduction

*Streptococcus dysgalactiae* (SD) causes a broad spectrum of human infections ranging from asymptomatic carriage via non-invasive soft tissue infections to life threatening conditions like necrotizing fasciitis and toxic shock syndrome (Brandt and Spellerberg, 2009). In the past decades there has been a significant increase in invasive infections in humans caused by SD in several geographical regions, and SD is currently among the most common pathogens detected in bloodstream infections in some countries (Couture-Cossette et al., 2018; UK Health Security Agency, 2022; Nevanlinna et al., 2023; Oppegaard et al., 2023).

SD is not a strict human pathogen, but capable of infecting a broad range of host species. It is recognized as a major cause of bovine mastitis, arthritis in swine and ovine lambs and necrotic ulcers in fish in aquaculture (Abdelsalam et al., 2010; Moreno et al., 2016; Zhang et al., 2018; Smistad et al., 2021). Moreover, SD is associated with a variety of infections in dogs, cats, and horses, underpinning the ecological versatility of this pathogen (Acke et al., 2015). At the same time, a phylogenetic diversity is evident within the SD taxon, and likely extends beyond the current delineation into the subspecies *Streptococcus dysgalactiae* subsp*. dysgalactiae* (SDSD) and *Streptococcus dysgalactiae* subsp*. equisimilis* (SDSE) (Ciszewski et al., 2016; Alves-Barroco et al., 2022). SDSD is, by definition, restricted to α-hemolytic group C strains, predominantly infecting cattle and sheep, whereas SDSE comprises all β-hemolytic strains (Vieira et al., 1998).

Penicillin remains a cornerstone in the treatment of infections caused by SD, and resistance to this antibiotic is exceedingly rare. Regarding second line alternatives, however, the situation is more alarming. Rising rates of macrolide, lincosamide and streptogramin (MLS) resistance have been noted, and in the United Kingdom MLS resistance is approaching 40% in SD isolates collected from humans (UK Health Security Agency, 2022). Even higher rates have been reported in bovine associated SD in China, as well as SD isolated from swine in South America (Moreno et al., 2016; Zhang et al., 2018). Moreover, increasing numbers of tetracycline resistant pyogenic streptococci are observed, and, as for the MLS resistance, this trend seems independent of host species (Abdelsalam et al., 2010; Ciszewski et al., 2016; Garch et al., 2020).

The main drivers for increasing antibiotic resistance are selection of resistant microbes by use and overuse of antibiotics, the possibility of horizontal genetic transfer of resistance traits between bacteria, and the spread of resistant bacteria between different geographic and ecological environments, both locally and globally. The complex ecological processes call for collaboration of multiple science fields in a “One health perspective” to approach and overcome emerging antibiotic resistance (McEwen and Collignon, 2018). However, our current knowledge on antimicrobial resistance in SD is predominantly based on studies limited to distinct host reservoirs, and data on dissemination of resistance determinants between ecological niches is scarce. Hence, there is a need for comparative studies on contemporary and spatially related isolates from different host species to investigate possible pathways for spreading of resistance traits.

We sought to explore antimicrobial resistance of SD in a One Health perspective and have examined clinically relevant SD isolates collected from various host species within a confined temporal and geographical setting. By dissecting phenotypic susceptibility patterns and whole genome sequences, we compared the resistomes associated with the different host specific reservoirs and attempted to elucidate possible routes for spread of resistance traits.

## Methods

### Bacterial isolates

All SD isolates identified in human blood cultures in Norway during 2018 were collected as part of the Norwegian surveillance program for antimicrobial resistance (NORM, 2019). SD isolates from bovine sources were randomly selected among isolates from bovine mastitis by TINE SA mastitis laboratory (Molde, Norway) in 2018. Additionally, all SD isolates from clinical samples from animals submitted to the Norwegian Veterinary Institute in 2018 and 2019, were included. Only one isolate per person and one isolate per animal flock or herd was included.

Species identification in the primary laboratories was based on colony morphology (hemolytic reaction on 5% sheep blood agar and colony size >0.5 mm after 24 h of incubation), serogroup specificity using rapid Lancefield agglutination test, and matrix-assisted laser desorption/ionization time-of-flight mass spectrometry (MALDI-TOF MS). All isolates identified as SD were submitted to either Haukeland University Hospital, Bergen, or Østfold Hospital Trust, Grålum, for susceptibility testing and genomic characterization.

### Susceptibility testing

All isolates were examined for susceptibility to benzylpenicillin, tetracycline, erythromycin, clindamycin, and trimethoprim-sulphamethoxazole according to the NORM protocol (NORM, 2019). Briefly, isolates were plated on Mueller-Hinton agar supplemented with defibrinated horse blood and β-NAD, and minimum inhibitory concentrations (MIC) were determined using MIC gradient strips. The Kirby-Bauer double disc diffusion method was used to assign the constitutive macrolide-lincosamide-streptogramin (cMLS), the inducible MLS (iMLS) and the macrolide (M) resistance phenotypes. Clinical breakpoints (version 14.0) set by the European Committee for Antimicrobial Susceptibility Testing (EUCAST) were used for interpretation of susceptibility.

### Whole genome sequencing

Whole Genome Sequencing of 119 SD of human origin as well as all 133 SD from animals was performed at Haukeland University Hospital on an Illumina 4,000 HiSeq system to produce 150 bp paired end reads, as previously described (Oppegaard et al., 2017). The remaining 155 human isolates were sequenced at Østfold Hospital Trust by the Ion Torrent technology on an Ion S5XL system as previously described (Kaci et al., 2023).

### *In silico* analysis

For data generated on the Illumina HiSeq system, reads were trimmed with Trimmomatic v0.39 (Bolger et al., 2014). For Ion Torrent generated data, reads were processed with the incorporated S5 software plug-ins. All trimmed reads from the sequenced isolates were *de novo* assembled by SPAdes v5.14 (Bankevich et al., 2012). Genome annotation was accomplished using RAST v1.073 (Aziz et al., 2008). Species identity was confirmed by 16S rDNA analysis. A core genome single-nucleotide polymorphism phylogeny was generated by CSI Phylogeny at the Center for Genomic Epidemiology^1^ using default settings and the SDSE type strain NCTC13762 as a reference. The resulting maximum likelihood phylogenetic tree was visualized and annotated using the Interactive Tree of Life platform, iTol v6 (Letunic and Bork, 2021).

Multilocus sequence typing (MLST) of the isolates was performed using the MLST 2.0 software available at the CGE webpage. The *emm*-database at the Centers for Disease Control and Prevention webpage was used to determine the *emm*-types.^2^ A minimum spanning phylogenetic tree using MLST types was constructed using the Phyloviz online tool,^3^ using triple-locus variant limitation for clustering.

RESfinder was used to screen for the presence of resistance genes (Florensa et al., 2022). Geneious Prime v 2022.2 was used to inspect the genetic context of the resistance genes, and screen for known mobilization genes from streptococcal mobile genetic elements using a database adapted form CONJdb.^4^ BLASTn was used to search for closest matches to putative mobile elements.

There are no validated methods for genotypic distinction between the two subspecies SDSE and SDSD. In accordance with the phenotypic definition proposed by Vieira et al. (1998), we defined SDSD *in silico* as genomes harboring the Lancefield group C-antigen operon, lacking the streptolysin S operon (corresponding to an α- or nonhemolytic reaction on blood agar), and lacking the streptokinase gene (inferring that streptokinase activity on human plasminogen does not occur). All other genomes were classified as SDSE.

### Statistical analysis

Differences in resistance rates between the host associated SD populations were tested for statistical significance by Fisher’s exact test. A two-sided *p*-value <0.05 was considered significant. Due to the low number of isolates available from some host associated populations, the data were pooled into isolates derived from humans, companion animals (dog and horse) and livestock (cow, sheep, swine) for statistical analysis.

## Results

A total of 407 SD isolates were included in the study (Supplementary Table S1). Of these, 274 were isolated from human blood cultures in 2018, constituting all SD isolates registered in the national surveillance program this year (NORM, 2019). Among the 133 animal associated isolates, 97 originated from livestock, including cattle (*n* = 74), sheep (*n* = 11) and swine (*n* = 12). The remaining 36 isolates were from dogs (*n* = 20) and horses (*n* = 16).

### Whole genome sequencing and phylogenetic analyses

The draft genomes of the 407 SD isolates had an average assembly length of 2.10 Mb, GC content of 39.3%, 2,100 protein encoding genes, and a coverage of approximately 220x. Based on whole genome sequencing analysis, 83 out of 85 isolates from bovine and ovine sources were classified as SDSD, whereas all other isolates belonged to the subspecies *equisimilis*. This phylogenetic delineation was in line with the phenotypic species identification.

**Figure 1:**
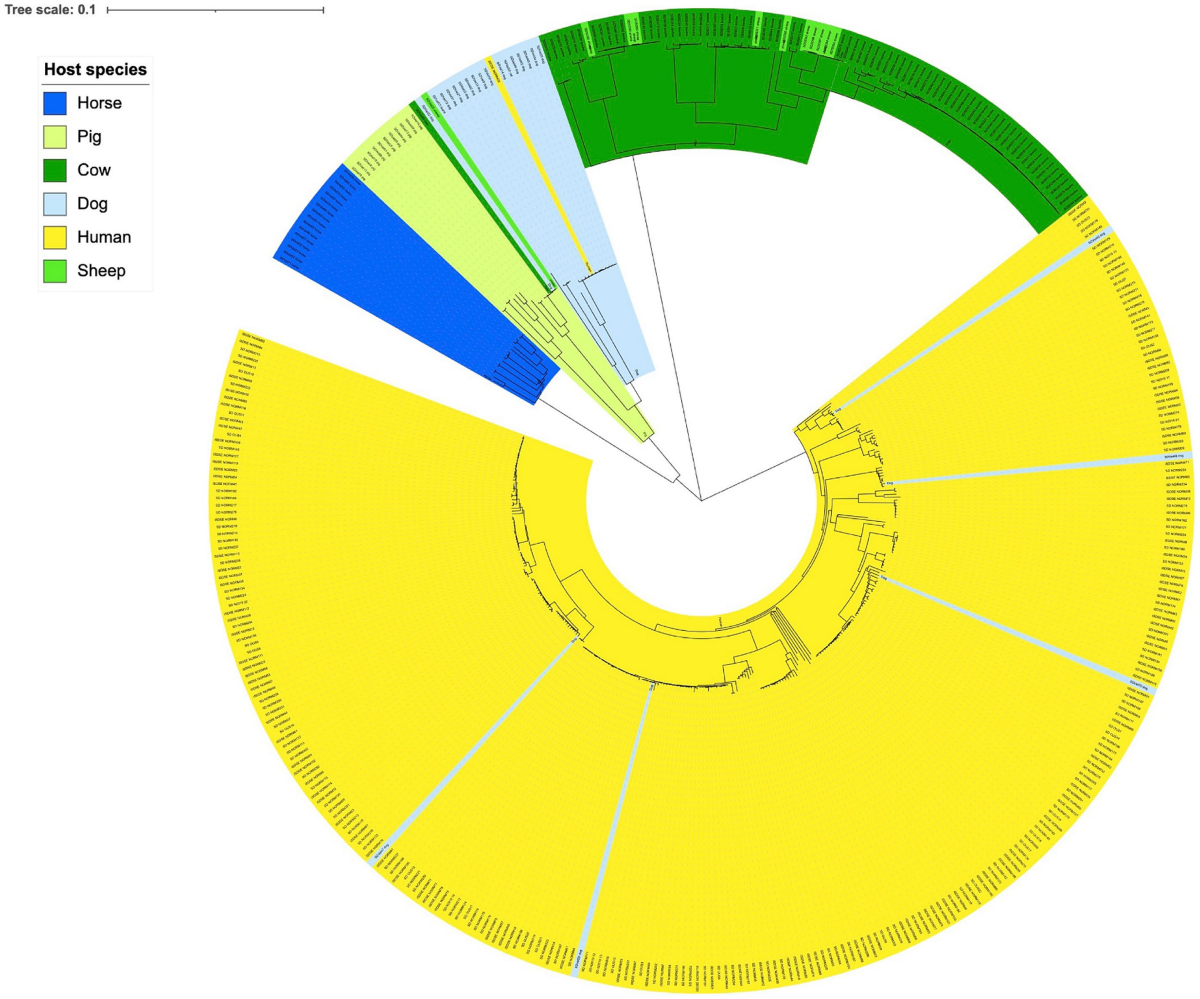
Genetic relationship between *Streptococcus dysgalactiae* isolates from different host species. Scale indicates substitutions per site. The phylogenetic tree is constructed based on a core genome single nucleotide polymorphism alignment using a maximum likelihood method.

Phylogenetic analysis delineated the isolates largely in accordance with host species (Figure 1). A notable exception was the distinct cluster corresponding to SDSD, where the isolates derived from bovine and ovine hosts were phylogenetically inseparable. The large clade of human associated isolates was clearly demarcated from the SD isolated from different animals. Nevertheless, indications of SD isolates crossing species barriers were observed. One isolate obtained from a human blood culture clustered phylogenetically with dog associated isolates. Inversely, five isolates obtained from dogs were found scattered in the cluster of isolates from humans, comprising 25% of all dog associated isolates. The minimum spanning tree based on MLST types was congruent with the single nucleotide polymorphism phylogenetic tree (Supplementary Figure S1).

### Phenotypic susceptibility testing

**Figure 2:**
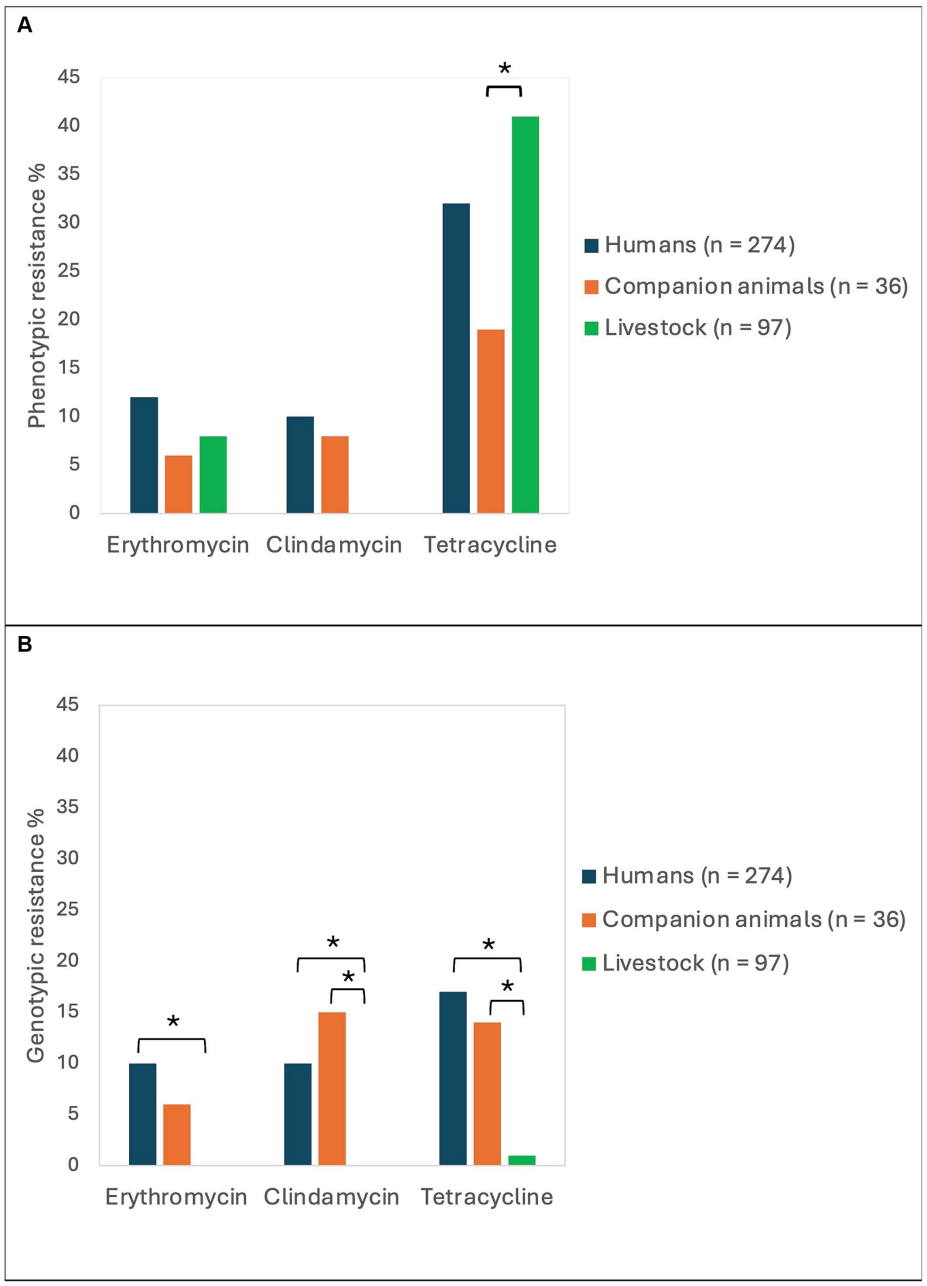
Antimicrobial resistance rates in *Streptococcus dysgalactiae* isolates from different host groups. The figure displays the prevalence of phenotypic **(A)** and genotypic **(B)** resistance to erythromycin, clindamycin, and tetracycline among *Streptococcus dysgalactiae* isolates procured from humans, companion animals and livestock animals. Asterisks mark significant differences in antimicrobial resistance levels.

The phenotypic antimicrobial resistance rates were relatively similar among isolates derived from humans, companion animals and livestock (Figure 2A). The only significant difference was a higher tetracycline resistance rate in SD from livestock (41%) compared to SD from companion animals (19%, *p* = 0.02). Resistance to erythromycin or clindamycin (MLS-resistance) was detected in 33 (12%), 3 (8%) and 8 (8%) of the isolates obtained from humans, companion animals and livestock, respectively. Of note, MLS-resistance in companion animals was derived from isolates from dogs only, and all MLS-resistance among livestock was detected in bovine associated isolates. All isolates in the study were susceptible to benzylpenicillin and trimethoprim-sulfamethoxazole.

### Resistome analysis

Whole genome sequences of all the included bacterial strains were screened for antimicrobial resistance genes (Table 1). In total, resistance genes were detected in 70 (26%), 9 (25%), and 2 (2%) of the isolates derived from humans, companion animals and livestock, respectively. We almost exclusively detected genes encoding resistance to either tetracyclines or MLS-antibiotics, and *tet*(M), *tet*(O) and *erm*(A) were the most abundant. However, distinct host associated resistance gene profiles were noted; *erm*(A) accounted for 82% of the MLS resistance in human associated isolates, whereas only *erm*(B) and *lsa*(C) were identified in MLS resistant SD from companion animals. Moreover, the rates of genotypic resistance to macrolides, clindamycin and tetracycline were all significantly lower among isolates procured from livestock than from humans and companion animals (Figure 2B).

**Table 1:** Genotypic resistance found in isolates of *Streptococcus dysgalactiae* of different host origin.

	Resistance gene	Humans (*n* = 274)	Companion animals (*n* = 36)	Livestock (*n* = 97)	Total
Tetracycline	*tet*(M)	29 (11%)	4 (11%)	0	33
	*tet*(O)	16 (6%)	1 (3%)	1 (1%)	18
	*tet*(T)	1 (< 1%)	0	0	1
	*tet*(W)	1 (< 1%)	0	0	1
MLS	*erm*(A)	23 (8%)	0	0	23
	*erm*(B)	2 (1%)	2 (6%)	0	4
	*erm*(T)	1 (<1%)	0	0	1
	*lsa*(C)	1 (<1%)	3 (8%)	0	4
	*mef*(A)	1 (<1%)	0	0	1
Other	*ant6Ia*	0	1 (3%)	2 (2%)	3
	*dfrF*	1 (< 1%)	0	0	1
	*cat*	1 (< 1%)	0	0	1

MLS, macrolide, lincosamide and streptogramin antibiotics. Numbers in parenthesis represent percentage of total number of isolates in the host category group.

**Figure 3:**
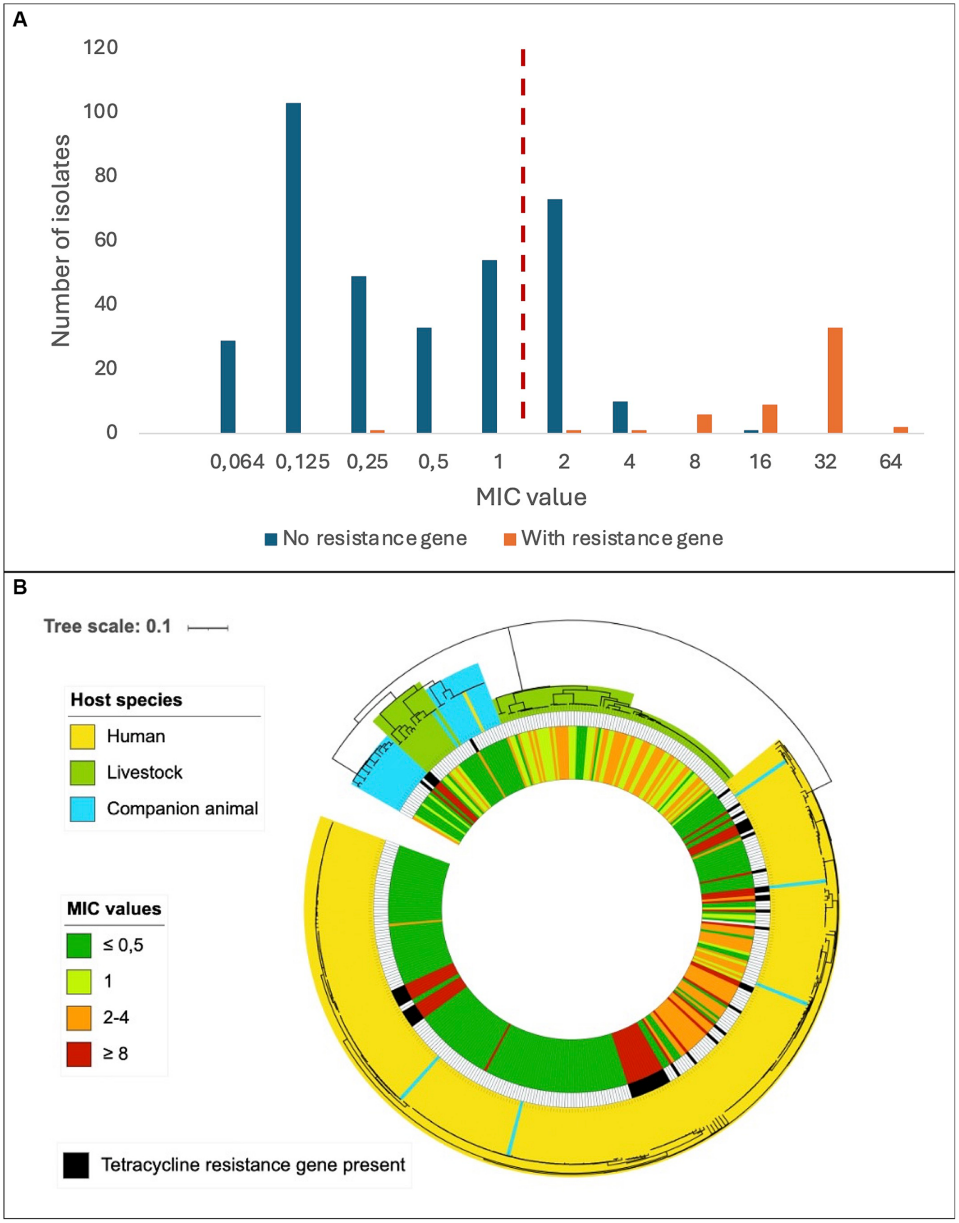
Distribution of tetracycline resistance in *Streptococcus dysgalactiae* isolates. **(A)** Minimum inhibitory concentrations of tetracycline among *Streptococcus dysgalactiae* isolates with or without a known tetracycline resistance gene. The dashed, red line represents the current EUCAST susceptibility breakpoint. **(B)** Phylogenetic tree of *Streptococcus dysgalactiae* isolates indicating host species (outer circle), presence of known tetracycline resistance gene (middle circle) and minimum inhibitory concentration of tetracycline (inner circle). Scale indicates substitutions per site.

The predicted genotypic resistance rates were substantially lower than the observed phenotypic resistance rates, particularly for tetracycline. Exploring this discrepancy, we found that the overall distribution of the tetracycline MIC values in our SD population appeared to be trimodal, and only the cluster with the highest MIC values correlated with isolates harboring genes encoding resistance to tetracycline (Figure 3A). The central cluster was intersected by the EUCAST tetracycline breakpoint, but the isolates lacked identifiable validated resistance genes. This resistance gene negative, low-grade resistant population included 39 of 40 livestock associated SD isolates with reduced susceptibility to tetracycline, but also comprised a distinct phylogenetic cluster of human associated strains (Figure 3B).

Incongruence between phenotypic and genotypic resistance traits was also observed for other antimicrobial agents. All eight erythromycin resistant livestock associated isolates had MIC values just above the susceptibility breakpoint, and none of them harbored identifiable resistance genes. An identical pattern was observed for 7 of 33 human associated SD isolates displaying reduced susceptibility to erythromycin.

Inversely, a few isolates had identifiable resistance genes but displayed phenotypical susceptibility, including three isolates harboring *lsa*(C)-genes, and one strain with a truncated *tet*(M)-gene.

### Mobile genetic elements and resistance genes

Analyses of flanking sequences of the detected resistance genes revealed a location on mobile genetic elements (MGEs) in almost all cases (Table 2). The one exception to this was a *tet*(M) gene located on a contig with a flanking sequence too short to determine the location with certainty. Integrative conjugative elements (ICEs) were the predominant form of MGEs detected, but the *erm*(T) gene was carried on a small *p5580*-like plasmid, and one dog isolate harbored a bacteriophage carrying an *erm*(B) gene. The major vector for MLS resistance was MGEs belonging to the ICE*Sp2905* family. These ICEs harbored 85% of the MLS resistance genes, including *lsa*(C) genes in both human and animal associated isolates. Nevertheless, ICE*Sp2905* elements in strains from different host sources displayed less than 95% sequence similarity based on core ICE conjugation genes.

**Table 2:** Antimicrobial resistance genes and their associated mobile genetic elements among human (H) and animal (A) associated isolates of *Streptococcus dysgalactiae*.

Resistance			Mobile genetic element											
Phenotype	Gene	*N*, total	*Sp2905*		*Sa2603*		*Tn916*		*Tn6944*		*Tn5801*		Other	
			H	A	H	A	H	A	H	A	H	A	H	A
Tetracycline resistance	*tet*(M)	33					13	1	14		1	3	1^a^	
	*tet*(O)	18	15		1	2								
	*tet*(T)	1	1											
	*tet*(W)	1	1											
MLS resistance	*erm*(A)	23	23											
	*erm*(B)	4				1	1						1^b^	1^c^
	*erm*(T)	1											1^d^	
	*lsa*(C)	4	1	3										
	*mef*(A)	1	1											

Data presented as number of isolates harboring the resistance gene. MLS; macrolide, lincosamide and streptogramin antibiotics. ^a^Unknown location. ^b^Tn6218. ^c^Bacteriophage. ^d^Plasmid.

On a similar note, the *tet*(O) genes, giving resistance to tetracycline, were located on ICEs belonging to the ICE*Sp2905* and ICE*Sa2603* family, but were distinctly associated with SD from human and animal sources, respectively. The location of *tet*(M) was more diverse, but the two major vectors in human associated SD isolates were the ICEs *Tn916* and *Tn6944*.

**Figure 4:**
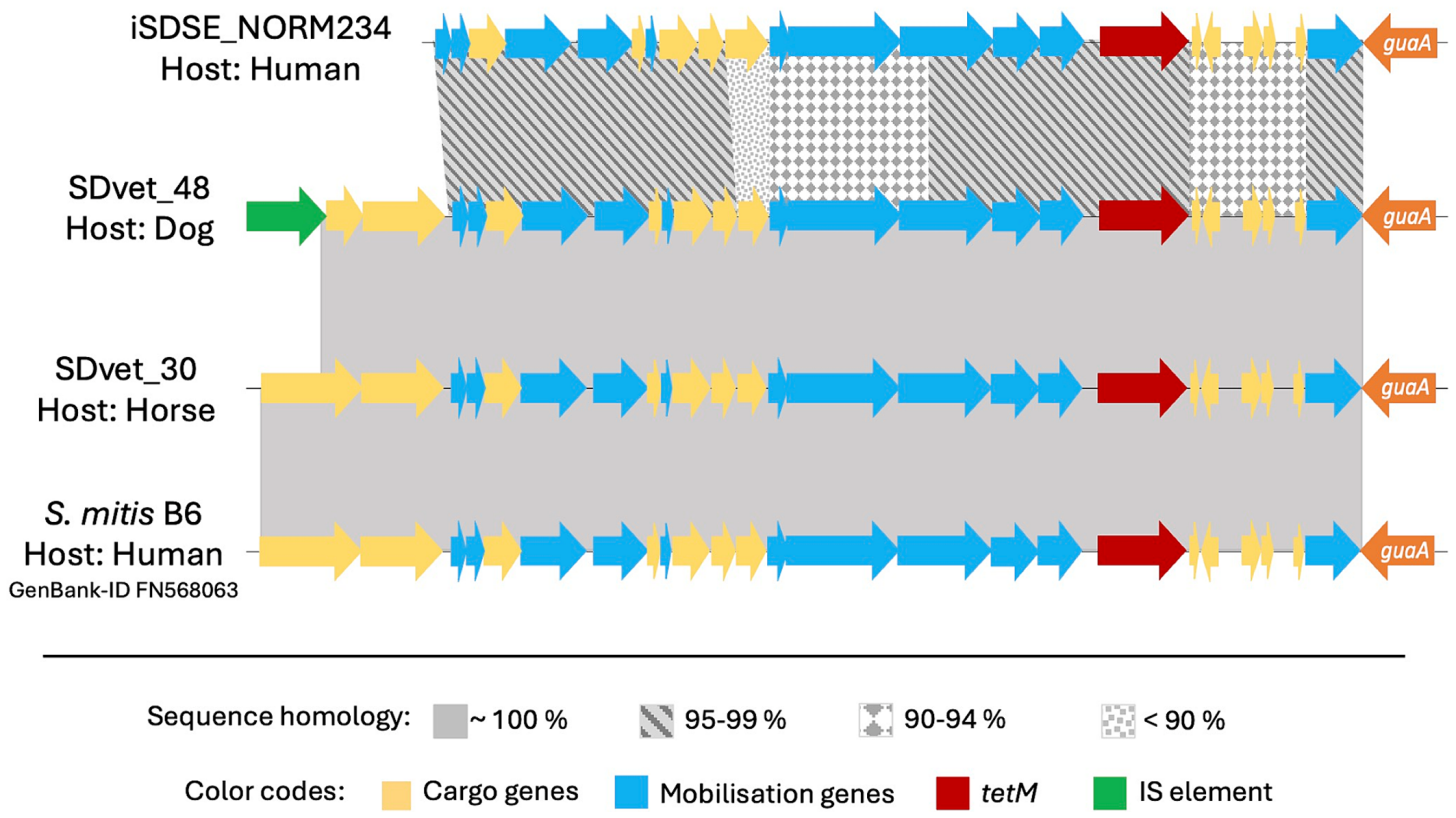
Comparative analyses of the *tet(M*) carrying mobile genetic element *Tn5801* in different bacteria. *Tn5801* was in all cases studied found integrated immediately downstream to the chromosomally located gene *guaA*. Among the *Streptococcus dysgalactiae* isolates included in this study, *Tn5801* was present in four: one from a human, one from a dog and two from horses. The *Tn5801* in the animal isolates had an inter-sequence homology close to 100%, while the human sequence was more divergent. This finding was not in line with the phylogenetic relationship of the bacterial isolates themselves, of which the isolate from the dog was more closely linked to the human isolate than to the horse isolates. By BLAST search we found *Tn5801* also to be present in other species than *S. dysgalactiae*, here represented by an element located in an invasive human isolate of *S. mitis*, which interestingly is a closer match to the mobile genetic element in *S. dysgalactiae* of animal than of human origin.

The element *Tn5801* was the most common harboring *tet*(M) in SD isolated from animal hosts. *Tn5801* is divided into type A and B, where the type B variant is lacking two genes at the beginning of the element (León-Sampedro et al., 2016). Two isolates from horses and one from a dog carried the type A variant of *Tn5801*, whereas one human associated isolate harbored the type B. Notably, the *Tn5801* element in the dog associated isolate SDVet48 clustered phylogenetically to the horse associated elements (Figure 4), even though the bacterial isolate itself phylogenetically resided among human isolates. By BLASTn search, both variants of the *Tn5801* elements were detected in a range of streptococcal, enterococcal and staphylococcal strains, isolated from both human and animal sources. Several of these showed more than 99% overall sequence homology to the type A and B *Tn5801* elements detected in our isolates.

## Discussion

To our best knowledge, this is the first comparative analysis of antimicrobial resistance patterns in contemporary *Streptococcus dysgalactiae* (SD) isolates in a One Health perspective. Overall, we found that direct transmission of bacterial strains or genetic content between different ecological niches appears to be infrequent. This is underpinned by the observed host specialization among the SD isolates and predominantly a difference in the genetic recipe for resistance, inferring different origins of the expressed traits.

A delineation of SD populations in accordance with host species has also been reported previously (Porcellato et al., 2021; Alves-Barroco et al., 2022). Porcellato et al. found that SD isolates harbored several host specific virulence factors and appeared to have evolved through genetic exchange with other bacterial species residing within their ecological niche (Porcellato et al., 2021). Segregation into host adapted phylogenetic lineages is also seen in studies of *Streptococcus agalactiae* and *Staphylococcus aureus*, and such niche specialization potentially represents a barrier to cross-species transmission (Maeda et al., 2020; Matuszewska et al., 2020). Supporting this, epidemiological collections of whole genomes of SD isolates from Japan, Canada and Denmark all reveal a very low frequency of animal associated MLST-profiles among SD isolated from humans, suggesting that zoonotic transmission of this pathogen is rare (Lother et al., 2017; Rebelo et al., 2022; Shinohara et al., 2023).

Despite the apparent transmission barrier conferred by niche adaptation, we found evidence of some level of cross-species exchange of SD isolates, predominantly between humans and companion animals. This is not surprising considering the close contact that often exists between humans and their dogs, and to a certain degree also between humans and horses. Similarly, Pinho et al. found that two SD isolates from dog and horse, respectively, were clustered together with human isolates in a phylogenetic study based on MLST analysis (Pinho et al., 2016). An SD isolate with the same MLST sequence type as the dog isolate to which it is referred was sampled from a boy living in the same household as the dog, reinforcing the hypothesis of a cross-species transmission event (Schrieber et al., 2014). In Singapore, an SD isolate phylogenetically resembling piscine associated SD strains was identified in a skin infection in a fish handler (Koh et al., 2009). Taken together, these findings indicate that cross-species transmission does occur, but predominantly in situations with prolonged or extensive exposure, such as between humans and their companion animals. Moreover, transmission events in a human to animal direction appear to be more common than the opposite, but the numbers are too small to draw firm conclusions.

We found highly diverging resistomes in SD isolates of human and animal origin, including both resistance genes and their associated MGEs, inferring limited genetic exchange between the host associated populations. Nevertheless, we observed an almost identical resistance element, *Tn5801*, in two SD isolates derived from a dog and a horse, respectively (Figure 4). Moreover, a highly similar element from a human associated *Streptococcus mitis* isolate was deposited in GenBank, strongly supporting the presence of genetic transfer between different ecological niches. However, the resistance MGEs detected in human associated SD isolates predominantly displayed similarities to MGEs derived from other β-hemolytic streptococcal isolates from humans, including *S. agalactiae* and *S. pyogenes*. Thus, the bulk of conjugative transfer and transduction of resistance determinants likely occurs within the boundaries of the ecological niche.

Reports on antimicrobial resistance in SD are quite sparse and limited to SD infecting humans and cattle. Studies on antimicrobial susceptibility in SD from the past decade demonstrate MLS resistance rates varying from 1 to 48% among bovine associated SD (Zhang et al., 2018; Duse et al., 2021) and from 17 to 42% among invasive, human associated strains (Park et al., 2019; Rojo-Bezares et al., 2021). Regarding tetracycline resistance, available data from the same period has shown resistance rates varying in the ranges 33–100% and 30–56% for bovine and human associated isolates, respectively (Traverso et al., 2016; Zhang et al., 2018; Park et al., 2019; Shen et al., 2021). Compared to these numbers, our findings indicate a relatively low frequency of resistant SD strains of both human and animal origin in Norway, possibly reflecting the strict policy regarding the use of antibiotics in both human and veterinary medicine in our country. European surveillance data about veterinary antimicrobial consumption obtained from the European Medicines Agency shows an at least tenfold higher consumption of all antibiotics relevant to this study in most European countries compared to Norway (EMA, 2024).

We observed a substantial incongruence between phenotypic and genotypic susceptibility rates for tetracycline, particularly in bovine associated isolates. In January 2023, EUCAST lowered the MIC breakpoint for tetracycline resistance in SD by merging the “I” (susceptible, increased exposure) category into the “R” (resistant) group. This change had a great impact on our results, doubling the number of strains entering the resistant category relative to earlier versions of the EUCAST clinical breakpoint table. Notably, most of these low-grade resistant strains lacked identifiable validated resistance genes. A MIC distribution intersected by the current EUCAST breakpoint was also reported in a recent Scandinavian study examining oxytetracycline-susceptibility among 231 SD isolates of bovine origin (Jensen et al., 2024). They found a uniform distribution with a proposed tentative epidemiological cut off (TECOFF) of 8 mg/L, which is three dilution steps above the breakpoint. Retrospectively applying the current breakpoint to previously published reports on tetracycline susceptibility in bovine associated SD, a tetracycline MIC distribution encircling the novel breakpoint was observed also in studies from Canada, New Zealand, and Europe, suggesting that this is a widespread feature in this species (McDougall et al., 2014; Cameron et al., 2016; Garch et al., 2020). In the present study a low-grade tetracycline resistant subpopulation was also evident among SD isolates of human origin (Figure 3B), indicating that this phenomenon is not limited to isolates of bovine origin, nor related to the distinction between the two subspecies SDSE and SDSD.

Breakpoints intersecting defined bacterial populations is generally avoided by EUCAST, as inherent analytic variations in the susceptibility testing makes the susceptible/resistant categorization unreliable. The observed high proportion of SD isolates displaying tetracycline MIC values encircling the breakpoint could either reflect a breakpoint poorly adapted to the SD wild type or be the result of a so far unrecognized mechanism of low-grade resistance. The trimodal distribution of the low-grade tetracycline resistant strains in our material could infer the latter, and the genetic basis for this phenomenon should be subjected to scrutiny.

In our study, we have included SD from a relatively large selection of host species, which entail a multifaceted base for comparative studies. The collection of strains from all hosts from within the same temporal and geographical delimitated setting is also a strength of the study, enabling a real time comparison of SD in a One Health perspective.

A limitation of the study is the low prevalence of antimicrobial resistance in Norway, potentially underestimating the extent of cross-species transmission. Antimicrobial resistance in streptococci in a One Health perspective should therefore be explored also in regions with higher rates of antimicrobial resistance. Moreover, analyzing pooled resistance rates for companion animals and livestock does not reflect the phylogenetic diversity within the SD taxon, and could potentially obscure significant differences between these ecological niches. Another potential limitation is the use of MIC gradient strips instead of disc diffusion or broth dilution. Nevertheless, a large proportion of our isolates was also examined by disc diffusion, and the results were congruent (Supplementary Table S1). A potential confounder is the delimitation of included human associated strains to exclusively bloodborne isolates, whereas the animal associated strains predominantly are from non-invasive infections. Nevertheless, national surveillance data on antimicrobial susceptibility in non-invasive human associated isolates of SD from 2018 does not reveal major differences between invasive and non-invasive strains (NORM, 2019). Lastly, we only examined isolates from a confined temporal context, and dissemination of resistance traits over time could not be evaluated. Longitudinal collection of isolates from asymptomatic carriers in contemporary and spatially related animal and human populations would be interesting. However, the execution of such an investigation probably would entail ethical challenges regarding sampling procedure on healthy animals.

In conclusion, we found a phylogenetic delineation of SD strains in line with host adapted populations and niche specialization. Moreover, the resistome differed significantly between SD in these host associated groups both regarding the repertoire of circulating resistance genes and their associated mobile gene elements. Our findings indicate that direct transmission events of strains or genetic elements carrying resistance genes between SD from different ecological niches are rare in our geographic region.

## Data availability statement

The datasets presented in this study can be found in online repositories. The names of the repository/repositories and accession number(s) can be found here: BioProject, accession number PRJEB74563, available at NCBI (https://www.ncbi.nlm.nih.gov/bioproject/?term=PRJEB74563).

## Ethics statement

The studies involving humans were approved by Regional Committee for Medical Research Ethics in Western Norway (2021/63132). The studies were conducted in accordance with the local legislation and institutional requirements. The human samples used in this study were acquired from a by-product of routine care or industry. Written informed consent for participation was not required from the participants or the participants’ legal guardians/next of kin in accordance with the national legislation and institutional requirements. Ethical approval was not required for the study involving animals in accordance with the local legislation and institutional requirements because samples from animals were collected from sick animals for diagnostic purposes by veterinarians in clinical practice, which does not require ethical approval.

## Author contributions

MG: Data curation, Formal analysis, Investigation, Visualization, Writing – original draft, Writing – review & editing. SS: Conceptualization, Funding acquisition, Writing – review & editing. AS: Writing – review & editing. BK: Conceptualization, Project administration, Writing – review & editing. AK: Writing – review & editing. CJ: Writing – review & editing. HJ: Writing – review & editing. OO: Conceptualization, Data curation, Investigation, Methodology, Project administration, Supervision, Writing – review & editing.

## Members of the Norwegian Study Group on *Streptococcus dysgalactiae*

Aasmund Fostervold, Department of Clinical Microbiology, Stavanger University Hospital, Stavanger, Norway; Aleksandra Jakovljev, Department of Microbiology, St. Olav’s University Hospital, Trondheim, Norway; Nadine Durema Pullar, Department of Clinical Microbiology, Vestre Viken Hospital Trust, Bærum, Norway; Åshild Marvik, Department of Microbiology, Vestfold Hospital Trust, Tønsberg, Norway; Einar Nilsen, Department of Microbiology, Møre and Romsdal Hospital Trust, Ålesund, Norway; Fredrik Müller, Department of Microbiology, Oslo University Hospital, Oslo, Norway; Ghantous Milad Chedid, Laboratory for Clinical Microbiology, Fonna Hospital Trust, Haugesund, Norway; Gunnar Skov Simonsen, Department of Microbiology and Infection Control, University Hospital of North Norway, Tromsø, Norway; Elisabeth Sirnes, Department of Microbiology, Division of Medicine, District General Hospital of Førde, Førde, Norway; Roar Magne Bævre-Jensen, Department of Clinical Microbiology, Vestre Viken Hospital Trust, Drammen, Norway; Sandra Åsheim, Unit for Clinical Microbiology, Norland Hospital Trust, Bodø, Norway; Ståle Tofteland, Department of Clinical Microbiology, Sørlandet Hospital Trust, Agder, Norway; Rolf-Arne Sandnes, Department of Clinical Microbiology, Innlandet Hospital Trust, Lillehammer, Norway; Truls Michael Leegaard, Department of Microbiology and Infection Control, Akershus University Hospital, Lørenskog, Norway.

## Footnotes

^1^ https://www.genomicepidemiology.org/

^2^ www.cdc.gov/streplab

^3^ online.phyloviz.net

^4^ conjdb.web.pasteur.fr
