# Supplementary material for: Antimicrobial resistance patterns in Streptococcus dysgalactiae in a One Health perspective
Source: Front Microbiol. 2024 Jun 6;15:1423762. doi: 10.3389/fmicb.2024.1423762 (PMC11348040; doi:10.3389/fmicb.2024.1423762)
Supplement: Supplementary file 1 [file Presentation_1.PPTX]

## Slide 1
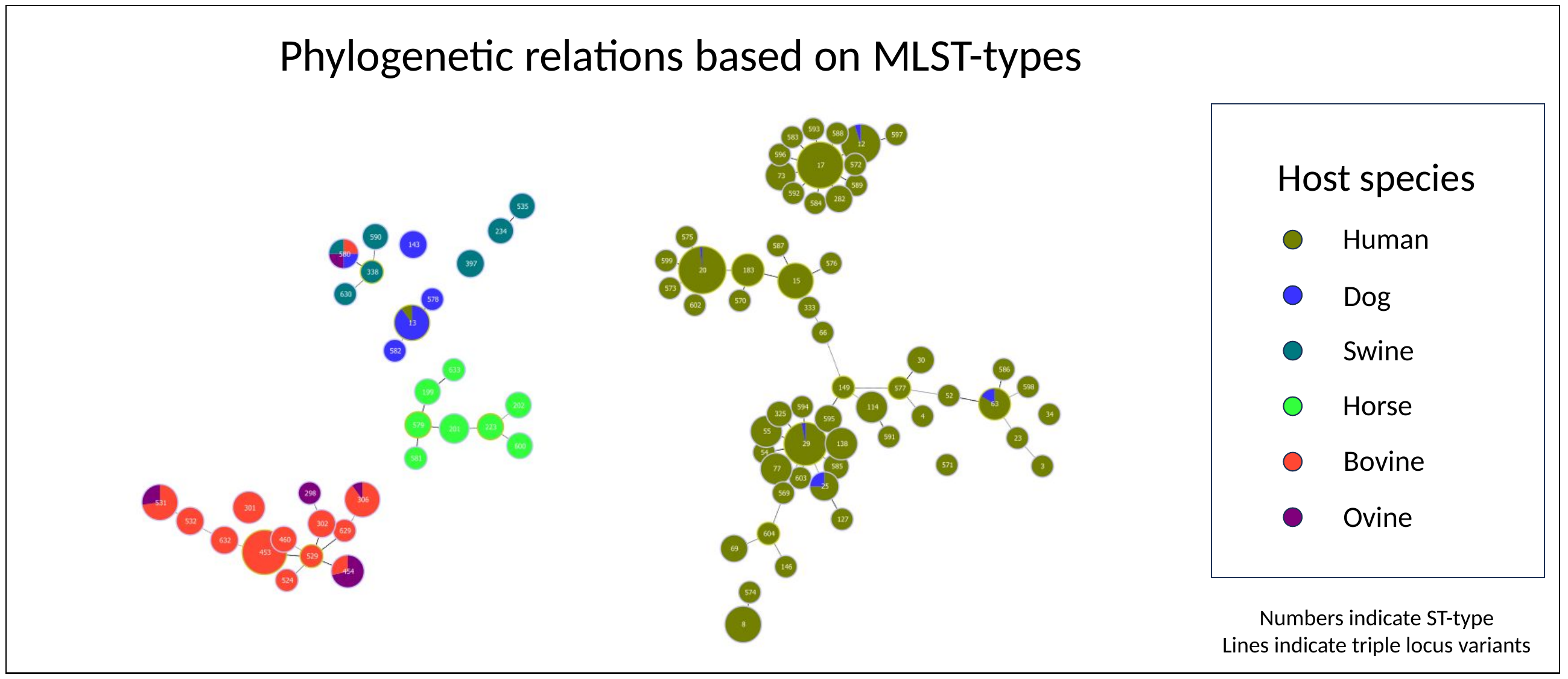

Phylogenetic relations based on MLST-types
Host species
Human
Dog
Swine
Horse
Bovine
Ovine
Numbers indicate ST-type
Lines indicate triple locus variants
